# Risk of self-harm in patients with body dysmorphic disorder: a population-based cohort study

**DOI:** 10.3389/fpsyt.2025.1660960

**Published:** 2025-12-05

**Authors:** Wai Kwong Tang, Kelvin K. F. Tsoi, Terry Cheuk Fung Yip, Vivien Wei Jun Liew, Selina Kit Yi Chan

**Affiliations:** 1 Department of Psychiatry, The Chinese University of Hong Kong, Hong Kong, Hong Kong SAR, China; 2 Jockey Club School of Public Health and Primary Care, The Chinese University of Hong Kong, Hong Kong, Hong Kong SAR, China; 3 Stanley Ho Big Data Decision Analytics Research Centre, The Chinese University of Hong Kong, Hong Kong, Hong Kong SAR, China; 4 Department of Medicine and Therapeutics, The Chinese University of Hong Kong, Hong Kong, Hong Kong SAR, China

**Keywords:** body dysmorphic disorder, self-harm, risk, depression, suicidal, cohort study

## Abstract

**Objective:**

This study was conducted to determine whether individuals with body dysmorphic disorder (BDD) have an increased risk of self-harm behaviors compared with those without BDD.

**Methods:**

In this matched cohort study, we reviewed the electronic health records of all patients admitted for any reason to Hong Kong public hospitals between January 1, 1993, and December 31, 2022. We selected a cohort of 71 patients with BDD and a comparison cohort of 71 patients without BDD. Participants were followed until a diagnosis of self-harm, death from other causes, or the end of 2023, whichever occurred first. Cox proportional hazards regression models were used to assess the risk of self-harm since the onset of BDD.

**Results:**

During the 30-year study period, 2 (2.8%) individuals in the BDD group and 3 (4.2%) in the comparison group exhibited self-harm behavior. The proportion of individuals engaging in self-harm was similar between the groups (χ^2^ = 0.25, p = 0.62). The incidence of self-harm was 22.5 and 34.6 per 10, 000 person-years in the BDD and comparison groups, respectively. The adjusted hazard ratio for self-harm was 0.63 (95% confidence interval, 0.08–4.56) in the BDD group compared with the comparison group.

**Conclusions:**

BDD is not associated with an increased risk of self-harm. Future studies are needed to replicate our findings and further identify potential risk factors for self-harm in patients with BDD.

## Introduction

Body dysmorphic disorder (BDD) is a mental health condition characterized by an individual’s persistent preoccupation with one or more perceived flaws in their appearance that are either minor or imperceptible to others. This intense focus on perceived imperfections leads to feelings of embarrassment, shame, and anxiety, causing individuals to avoid social situations. According to the American Psychiatric Association, individuals with BDD obsess over their appearance and body image for hours each day. The *Diagnostic and Statistical Manual of Mental Disorders, Fifth Edition*, classifies BDD as part of the obsessive-compulsive spectrum because of the presence of obsessive thoughts, compulsive behaviors, and high levels of stress and avoidance behavior (1).

BDD affects approximately 2% of the general population (2), typically beginning in adolescence, a period when individuals are particularly sensitive regarding their appearance. BDD occurs in both men and women at similar rates across races and ethnicities (3). BDD is a chronic condition that is often under-recognized and under-diagnosed (4). Thus, many individuals with BDD do not receive the diagnosis or treatment they need. BDD is associated with substantial impairment in psychosocial functioning, leading to a poor quality of life (5). The persistent and intrusive focus on perceived physical flaws, combined with repetitive behaviors such as mirror checking, excessive grooming, skin picking, and seeking reassurance, results in considerable emotional distress (2). The stigma and shame associated with BDD frequently prevent individuals from seeking help, exacerbating their feelings of isolation and despair (6). Moreover, BDD often co-occurs with other mental health disorders, such as major depressive disorder and anxiety disorders (2), which may contribute to an increased risk of suicidal ideation in this population. Veale and Gledhill (7) indicated the importance of healthcare professionals being vigilant for signs of suicidality in individuals with BDD. They also highlighted the need for comprehensive treatment approaches that address the full spectrum of BDD symptoms and impairments, including suicidal ideation and attempts. Effective treatment includes cognitive-behavioral therapy, which has been found to be beneficial for BDD, and medications, such as selective serotonin reuptake inhibitors (8, 9).

The theoretical foundation for understanding the increased risk of self-harm in BDD is primarily based on a cognitive-behavioral model, which posits that individuals with BDD experience distorted and excessive attention to perceived physical flaws, leading to negative self-appraisal and persistent anxiety (10, 11). These cognitive distortions generate a vicious cycle of rumination and maladaptive behaviors, such as mirror checking or camouflaging, which maintain and exacerbate distress (10–12). Additionally, environmental factors, including childhood trauma and peer victimization, significantly contribute to the development and perpetuation of negative body image and low self-esteem, further increasing vulnerability to self-harm (10, 12). Phenomenological perspectives add that the experience of the body as alien or betrayed can drive individuals toward extreme behaviors like self-injury to “correct” perceived defects, consistent with theories of self-discrepancy and existential distress (13, 14). Comorbid affective disorders such as anxiety and depression often intensify emotional dysregulation, making self-harm a maladaptive coping strategy (10, 11, 15).

Published data on suicide attempts or ideation among individuals with BDD are limited. A population-based survey of 45 patients with BDD reported that 22% had attempted suicide (16). Another population-based survey of 43 patients with BDD found that 7% had attempted suicide (17). A cross-sectional study of 16 psychiatric outpatients with BDD revealed a lifetime suicide attempt rate of 19%, which was comparable to that in individuals without BDD (18). A study using a convenience sample of 200 individuals with BDD suggested that 26%–28% had attempted suicide (19). Furthermore, in a cohort of BDD patients receiving mental health service, 50% had engaged in self-harm or attempted suicide at some point in their lives (20). Another cohort study found that patients with BDD had a 3-fold increased risk of intentional self-harm (21). In addition, a meta-analysis of 9 studies demonstrated that BDD was associated with an increased risk of suicide attempts (odds ratio [OR] = 3.3) ^2^. However, previous studies have several limitations, including a cross-sectional design (16–19); small sample sizes (18); recruitment of convenience samples or research participants (19); reliance on self-reported self-harm; and high levels of clinical, methodological, and statistical heterogeneity (2). The present study determined whether individuals with BDD have an increased risk of self-harm behaviors compared with those without BDD.

## Materials and methods

### Data source

In this matched cohort study, we used the electronic health records of all patients admitted to Hong Kong public hospitals for any reason between January 1, 1993, and December 31, 2022, sourced from the Hong Kong Clinical Data Analysis and Reporting System (CDARS). Data from the CDARS have been used in previous epidemiological studies, with demonstrated reliability (22). The system contains electronic health records from the Hong Kong Hospital Authority, a statutory body that manages all public hospitals serving the 7.7 million residents of Hong Kong. Hong Kong’s current healthcare system operates at three levels of care, namely primary, secondary, and tertiary, provided through both public and private sectors (23). Public healthcare services are managed by the Hospital Authority, which oversees all public hospitals. According to data from 2017, the Hospital Authority accounted for approximately 80% of inpatient visits and 30% of outpatient visits (24). The CDARS encrypts patients’ personal information to ensure privacy and provides researchers with anonymous identification numbers. The CDARS has been demonstrated to be a valid and reliable data source. A validation study found that the positive predictive value of fracture diagnoses in the CDARS was 96.8% (25). This study examines self-harm in BDD using CDARS data. Another study by the author investigated self-harm behaviors in Trichotillomania (Manuscript ID 1688488). The present study was approved by the Institutional Review Board of the Chinese University of Hong Kong (CREC 2020.707).

### BDD cases

We identified cases as individuals who received outpatient, emergency department, or inpatient care with a first-recorded diagnosis of BDD during the study period. For each case, the date of the initial BDD diagnosis was defined as the date when the follow-up started. BDD cases were identified using the International Classification of Diseases, Ninth Revision (ICD-9) code 300.72.

### Non-BDD comparators

To establish a comparable non-BDD cohort, we randomly selected individuals without a history of BDD and matched them by sex and age (at admission) with patients with BDD. A random month and day, along with the same index year as the matched patient’s diagnosis date, were assigned as the index date for the comparators. For all non-BDD comparators, follow-up began from the matched admission date.

### Covariates

For both the BDD and comparison groups, information on ethnicity, residential districts, and diagnoses of depression and bipolar disorders (ICD-9 code 296) was retrieved from the CDARS.

### Outcome measurement

All participants were followed until the occurrence of self-harm, death from any cause, or the end of 2023, whichever occurred first. Given that self-harm events are often under-reported in hospital administrative datasets due to stigma and challenges in determining intent (26), we adopted a broader definition of self-harm beyond the standard ICD-9 criteria. This approach included all self-injurious behaviors, both with and without suicidal intent, whether unintentional or intentional. The ICD-9 codes used to identify self-harm were E950–59 (self-harm) and E980–89 (self-harm, undetermined intent). Participants were followed from the date of BDD diagnosis (or the matched admission date for comparators) until the first record of self-harm, death from any cause, or December 31, 2023, whichever occurred first.

### Statistical analysis

The number and proportion of individuals who engaged in self-harm were calculated for both the BDD and comparator groups. Differences in the proportion of individuals who engaged in self-harm between the groups were determined using the χ^2^ test. Incidence curves were plotted using the Kaplan–Meier method to identify the temporal patterns of self-harm following the first-recorded diagnosis of BDD, and comparisons were made using the log-rank test. The incidence of self-harm was calculated as the number of self-harm events divided by the total follow-up period (per 10, 000 person-years). Cox proportional hazards regression models were used to calculate the hazard ratio (HR) and 95% confidence interval (CI) for the risk of self-harm after BDD onset, adjusting for age, sex, ethnicity, residential district, and diagnoses of depression and bipolar disorders. All statistical analyses were performed using STATA (StataCorp, College Station, TX, USA). A *p* value of <0.05 was considered statistically significant.

## Results

**Figure 1:**
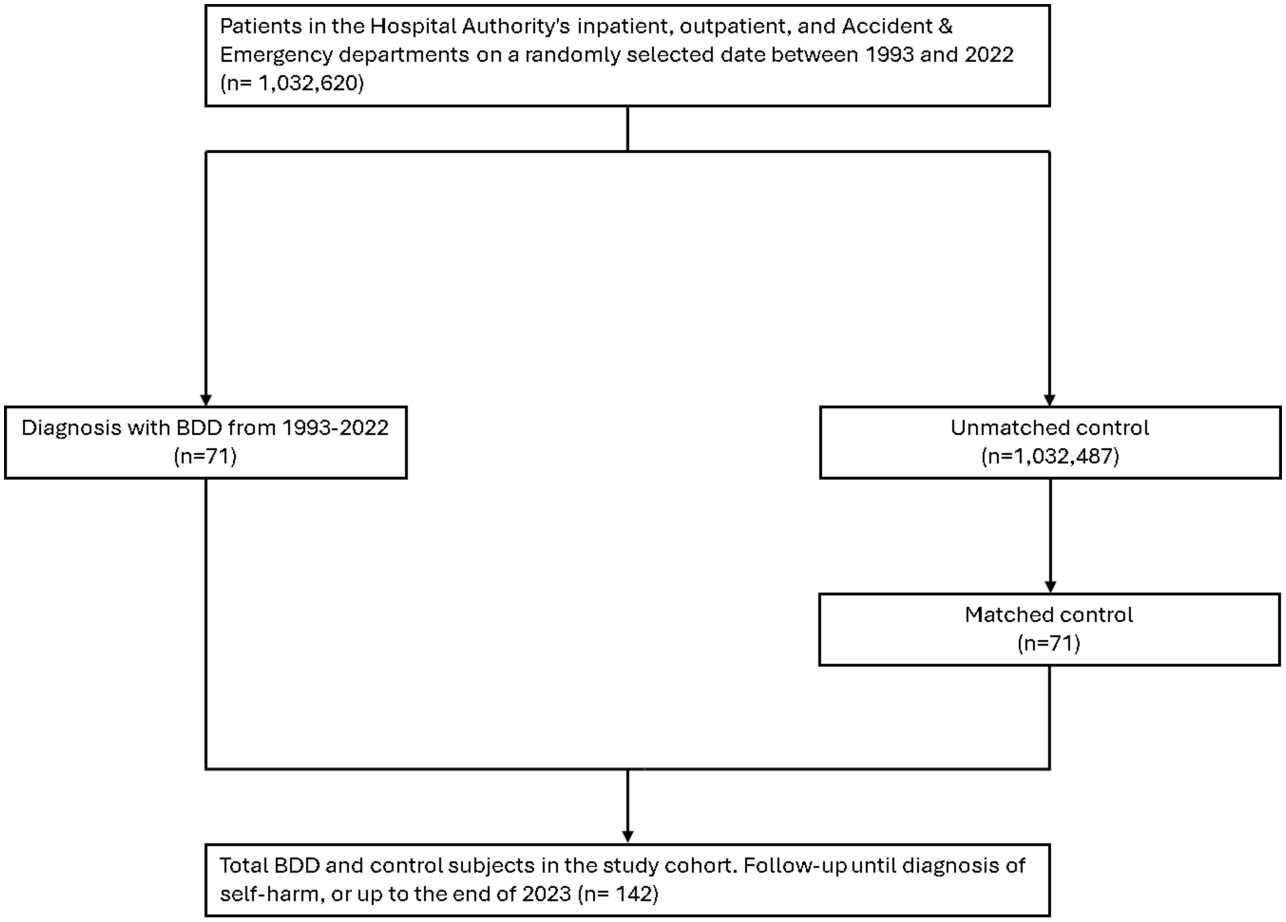
The flowchart of participant selection.

**Figure 2:**
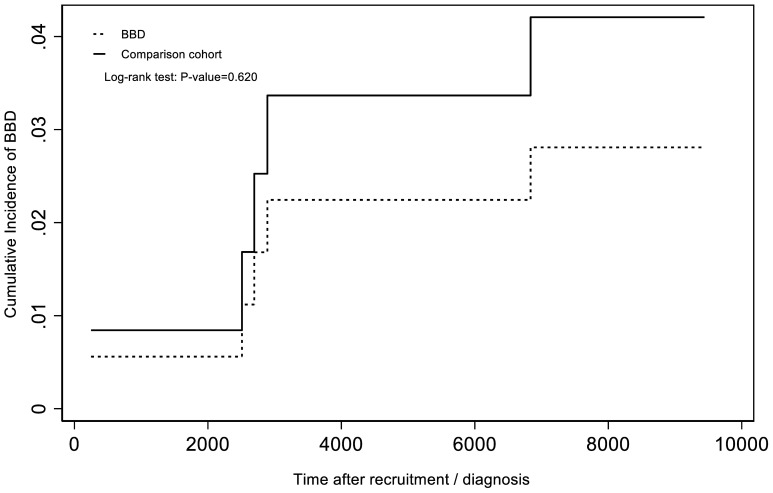
Kaplan-Meier analysis, adjusted for age, sex, ethnicity, residential district, and history of depression and bipolar disorders.

We selected a cohort of 71 patients with BDD and a matched comparison cohort of 71 individuals without BDD (**Figure 1**). The distributions of sex (52% women), ethnicity, age (22.6 ± 15.5 years), and residential district did not significantly differ between the BDD and comparison groups. The prevalence of depression or bipolar disorder was 14.1% in the BDD groups and 15.5% in the comparison group (**Table 1**). Over the 30-year study period, the incidence of self-harm was 22.5 and 34.6 per 10, 000 person-years in the BDD and comparison groups, respectively. The Kaplan–Meier analysis indicated that the incidence of self-harm was similar between the BDD and comparison groups (log-rank *p* = 0.6203; **Figure 2**). The unadjusted HR for self-harm in the BDD group compared with the comparison group was 0.64 (95% CI, 0.11–3.82). After adjustment for age, sex, ethnicity, residential district, and depression or bipolar disorders, the HR for self-harm was 0.63 (95% CI, 0.08–4.56) in the BDD group compared with the comparison group. The average follow-up period for self-harm was 9.7 ± 12.8 years in the BDD groups and 7.4 ± 0.5 years in the comparison group. The median time to self-harm (interquartile range [IQR]) was 9.7 years (0.7–9.7) in the BDD group and 7.4 years (6.7–7.4) in the comparison group.

**Table 1:** Demographic and clinical characteristics in the study cohort.

Demographic and clinical characteristics	BDD (n=71)	Comparison cohort (n=71)	P value ^a^
Gender			1.000
Female	37 (52.1)	37 (52.1)	
Male	34 (47.9)	34 (47.9)	
Age (mean ± SD)	22.6 ± 15.5	22.6 ± 15.5	1.000 ^b^
Ethnicity ^c^			0.127
Chinese	67 (94.3)	70 (98.6)	
Non-Chinese	1 (1.4)	1 (1.4)	
District of residence ^d^			
Kowloon	34 (47.9)	17 (23.9)	0.566
Hong Kong Island	19 (26.8)	38 (53.5)	0.499
New Territories	18 (25.4)	10 (14.1)	0.805
History of depression/bipolar disorder	10 (14.1)	11 (15.5)	0.813
Time to self-harm (years), mean (mean ± SD), median (IQR)	9.7 ± 12.8 9.7 (0.7-9.7)	7.4 ± 0.5 7.4 (6.9-7.4)	0.862

a Pearson chi-square test.

b t-test.

c 3 subjects had no ethnicity data.

d 1 and 3 subject had no district of residence data in BBD and comparison cohort respectively.

SD= standard deviation; IQR= interquartile range.

## Discussion

To the best of our knowledge, this study is the first to systematically investigate the risk of self-harm in patients following their first-recorded diagnosis of BDD. No significant difference in the incidence of self-harm was observed between the BDD and comparison groups, even after adjusting for diagnosed mood disorders.

In the present study, the risk of self-harm was similar between the BDD and comparison groups. In contrast, studies on obsessive-compulsive disorder (OCD) have reported an increased risk of suicide and suicide attempts in these individuals. For example, a population-based cohort study conducted in Sweden found that the HR for suicides was 4.9 among people with OCD compared with full siblings without OCD (27). Another population-based study conducted in Sweden reported that the OR for suicide attempts was 5.5 in patients with OCD compared with matched controls (28). Furthermore, a systematic review of epidemiological studies examining the association between OCD and lifetime suicide attempts in the general population found that individuals with OCD had a significantly higher odds of a lifetime suicide attempt (OR ranging from 1.6 to 9.9) than did those without OCD (29). Several factors may explain the negative findings in the present study, including potential under-reporting of self-harm in the BDD group, an increased risk of self-harm in the comparison group, and the younger age and lower prevalence of mood disorders in the BDD group. Methodological differences between the previous studies and the present study may also contribute to the differing results, such as focusing on completed suicides instead of suicide attempts (27), using unaffected siblings as controls (27), and not adjusting HRs for depression or bipolar disorder (28).

In the present study, 2.8% of patients engaged in self-harm; this figure is lower than the rate of suicide attempts (7% to 28%) reported in previous cross-sectional and cohort studies and meta-analysis (16–19, 21). One possible explanation for this discrepancy is that self-harm in the current study was identified through medical records, whereas other studies relied on self-reported data. Mild forms of self-harm that do not require medical attention may not be captured in the CDARS. In addition, self-harm episodes that occurred before the first diagnosis of BDD were excluded from the analysis.

Our study has several limitations. The sample was obtained from medical records in the CDARS, which does not include data from private hospitals, clinics, or general outpatient services. Thus, the comparators selected from hospital and outpatient clinic databases may have had a higher risk of self-harm than the general population, leading to an underestimation of HRs. However, our study design included an adequate comparison group that was matched by age and sex to minimize this bias.

We adopted a broad definition of self-harm that included deliberate self-injurious behaviors with suicidal intent, self-injurious actions without suicidal intent, and self-injurious actions that were unintentional or intentional. In a study examining the risk of self-harm among psychiatric patients in Hong Kong, sensitivity analysis revealed that excluding undetermined self-harm (ICD-9 codes E980–89) did not substantially affect the results (30). In addition, our dataset lacked information on the interventions and treatments provided to patients, which may have influenced the results. Only suicidal actions were recorded in the CDARS. Moreover, the prevalence of suicidal ideation is likely higher than the frequency of self-harm, potentially being under-reported in clinical practice.

In the present study, we investigated the association between the onset of BDD and subsequent self-harm behaviors. However, because clinical records prior to 1993 were unavailable, we could not confirm whether the first record in the database represented the actual first diagnosis of BDD. In addition, data on self-harm were derived only from medical records, which may have led to under-reporting. The prevalence of depression and bipolar disorder in both the BDD and comparison groups was very low, reflecting the general underdiagnosis of psychiatric disorders (31). This low rate of mood disorders in our study might have resulted in an underestimation of the impact of these conditions on the risk of self-harm.

Finally, self-harm is a complex behavior affected by multiple factors, including demographic, social, economic, cultural, psychological, and environmental components (32). However, because of limitations in our data source, we were unable to account for these risk factors. Ideally, information on sociodemographic factors (e.g., marital and employment status and education level) and potential confounders (e.g., physical comorbidities, smoking, and alcohol use) should be available and adjusted for in the analysis.

This study presents several key strengths that enhance understanding of self-harm risk in BDD compared to prior research. First, the matched cohort design controlled for confounding factors such as age and sex has ensured valid risk comparisons between BDD and non-BDD groups. Second, the population-based sample derived from general hospital electronic health records increased the generalizability of our findings. Third, the extensive 30-year follow-up period allowed for the capture of long-term risk patterns, while the comprehensive use of electronic health records minimized recall and selection biases common in clinical samples and enhanced the accuracy of self-harm event detection.

Clinically, although our findings suggest that BDD may not increase the risk of self-harm compared to controls, extensive evidence indicates that BDD is associated with substantial psychological distress and comorbid psychiatric disorders such as depression and anxiety (2), as well as elevated suicidal ideation and attempts among patients (7, 16–21). Therefore, comprehensive clinical assessment for self-harm and suicide risk is essential for individuals with BDD, especially given that up to 80% have reported suicidal thoughts and up to 28% have attempted suicide in broader samples (19, 33). Early identification and treatment remain critical, with cognitive behavioral therapy (CBT) tailored specifically for BDD and selective serotonin reuptake inhibitors (SSRIs) as first-line interventions shown effective in reducing symptom burden and associated risk behaviors (8, 9, 33). Engagement strategies, including motivational interviewing and psychoeducation to shift patients’ insight from physical to psychological understanding of symptoms, are also recommended (33). Clinicians should also monitor for high-risk behaviors such as “do-it-yourself” cosmetic procedures that may pose additional harm (33). Interventions targeting comorbidities such as depression and anxiety, regular risk assessments, and family involvement for support are advised to optimize psychosocial functioning and prevent self-harm (7, 33, 34). Finally, broader awareness campaigns within healthcare settings and communities are needed to destigmatize BDD and promote early referral and intervention (35).

## Conclusions

BDD is not associated with an increased risk of self-harm. Future research is needed to replicate these findings and further explore risk factors for self-harm in these patients.

## Data Availability

The data analyzed in this study is subject to the following licenses/restrictions: The data used in this study were obtained from the Clinical Data Analysis and Reporting System (CDARS) managed by the Hospital Authority (HA) in Hong Kong. Due to privacy and legal restrictions, data access is strictly controlled and limited to anonymized datasets, subject to HA approval. Requests to access these datasets should be directed to Hospital Authority, https://www3.ha.org.hk/data/Provision/ApplicationProcedure. Further inquiries can be directed to the corresponding author/s.
